# HIV late presentation and advanced HIV disease among patients with newly diagnosed HIV/AIDS in Southwestern China: a large-scale cross-sectional study

**DOI:** 10.1186/s12981-019-0221-7

**Published:** 2019-03-16

**Authors:** Xi Hu, Bingyu Liang, Chongxing Zhou, Junjun Jiang, Jiegang Huang, Chuanyi Ning, Jie Liu, Bo Zhou, Ning Zang, Jinzhen Lai, Rongfeng Chen, Yanyan Liao, Peijiang Pan, Xin Liu, Guanghua Lan, Xianwu Pang, Li Ye, Zhiyong Shen, Hao Liang

**Affiliations:** 1 Guangxi Key Laboratory of AIDS Prevention and Treatment, School of Public Health, Guangxi Medical University, Nanning, 530021 Guangxi China; 2 Guangxi Collaborative Innovation Center for Biomedicine, Life Sciences Institute, Guangxi Medical University, Nanning, 530021 Guangxi China; 3 Institute of HIV/AIDS Prevention and Control, Guangxi Zhuang Autonomous Region Center for Disease Control and Prevention, Nanning, 530200 Guangxi China

**Keywords:** Late presentation, Advanced HIV disease, HIV/AIDS, Southwestern China

## Abstract

**Objective:**

This study aimed to investigate the prevalence of HIV late presentation and advanced HIV disease and to identify the factors associated with HIV late presentation and advanced HIV disease among patients with newly diagnosed HIV/AIDS in the Guangxi Zhuang Autonomous Region, in Southwestern China.

**Methods:**

Patients with newly diagnosed HIV registered in the HIV surveillance system of Guangxi Centers for Disease Control between January 2012 and December 2016 were included in this study.

**Results:**

Of 45,118 newly diagnosed patients, 70.2% had late presentation, and 45.1% had advanced HIV disease. A higher prevalence of late presentation and advanced HIV disease was found in male heterosexuals and female people who use drugs (PWID). Heterosexuals (OR 2.11 [95% CI 1.90–2.34]) and PWID (OR 1.55 [95% CI 1.30–1.84]) had a higher risk of late presentation than men who have sex with men (MSM). Blood testing of the blood receivers (OR 1.75 [95% CI 1.36–2.26]) and diagnosed in hospital (OR 1.74 [95% CI 1.65–1.84]) had an increased risk of late presentation compared to those who diagnosis in voluntary counseling and testing (VCT). Heterosexuals (OR 2.86 [95% CI 2.51–3.27]), PWID (OR 2.23 [95% CI 1.83–2.71]), blood testing of the blood receivers (OR 1.58 [95% CI 1.29–1.94]) and diagnosed in hospital (OR 1.85 [95% CI 1.76–1.94]) were also independent risk factors associated with advanced HIV disease. Older age, lower level of education and being divorced or widowed were also associated with late presentation and advanced HIV disease.

**Conclusions:**

Late presentation and advanced HIV disease were very common among patients with newly diagnosed HIV in Guangxi, China during 2012–2016. Targeted programs are urgently required to reduce HIV late diagnosis in Guangxi, especially for male heterosexuals, PWID, and patients with characteristics such as older age, lower level of education, divorced or widowed.

## Introduction

The number of people living with human immunodeficient virus/acquired immune deficiency syndrome (HIV/AIDS) has steadily increased worldwide and reached 36.9 million in 2017 [1]. In China, by the end of 2017, a cumulative total of 758,000 individuals were reported to be HIV infected, with more than 100,000 HIV-positive patients reported in the Guangxi Zhuang Autonomous Region [2]. To effectively control the global HIV/AIDS epidemic, the Joint United Nations Programme on HIV/AIDS (UNAIDS) put forward a 90–90–90 target in 2013, aiming to 90% of people living with HIV aware about their status, treat 90% of these detected cases with antiretroviral therapy (ART), and achieve viral load suppression in 90% of those receiving treatment by 2020 [3]. However, at present, only 60% of people with HIV infection are aware of their infectious status worldwide [1], which, to a great degree, stands in the way of achieving the 90–90–90 target.

Timely initiation of ART has been considered as one of the most effective approaches to reduce the risk of HIV transmission. Early diagnosis of HIV is a crucial step to achieve the goal of early treatment [4–6]. Nevertheless, almost half of HIV-positive patients are late diagnosed worldwide [7]. In Europe, more than one-third of patients with HIV/AIDS are late diagnosis, resulting in delayed treatment [8]. In China, a study conducted at the national level showed that 58.8% of patients with newly diagnosed HIV from 2006 to 2012 were late diagnosed [9]. Another study indicated that the rate of advanced HIV disease in China ranged from 35.5% to 42.1% during 2010–2014 [10]. Compared with those with early diagnosis, the patients with late diagnosis were worse in terms of immune system at diagnosis [11, 12], and paid a higher cost for the therapy [13]. Moreover, late diagnosed cases may cause inadvertent HIV transmission before they are aware of their HIV infection status [14]. More importantly, late diagnosis is always associated with higher mortality and morbidity due to various opportunistic infections, especially tuberculosis, invasive bacterial, and fungal infections [15]. A retrospective study revealed that among patients with advanced HIV disease, 57% had opportunistic infections and the majority of them were diagnosed when they developed AIDS-defined illness [16].

The Guangxi Zhuang Autonomous Region, a province in western China, has the second highest HIV-infected reported cases in China, accounting for ~ 13% of total national HIV/AIDS cases. Even worse, the mortality among patients with HIV/AIDS in that region reached up to 34.9% by the end of 2017, which is far higher than the average of national level (24.0%). Late diagnosis is one of the important predictors of HIV/AIDS-related mortality [17]. However, information about HIV late diagnosis in Guangxi, in addition to the influencing factors, should be further explored. To date, only a few studies reported the situation in a county or a city in Guangxi [18]. In addition, even in other cities of mainland China, only a small number of studies have identified the factors associated with late HIV diagnosis in several cities [19–22]. Reducing HIV/AIDS epidemic is extremely urgent for Guangxi through some effective targeted prevention strategies. Therefore, the present study investigated the situation of late presentation (CD4 < 350/mm^3^, or AIDS-defining event regardless of CD4 count) and advanced HIV disease (CD4 < 200/mm^3^, or AIDS-defining event regardless of CD4 count) among patients with newly diagnosed HIV/AIDS. Also, the influencing factors, including demographic or socioeconomic variables associated with late presentation and advanced HIV disease, were analyzed.

## Methods

### Study population

All patients with newly diagnosed HIV/AIDS who registered in the HIV surveillance system of Guangxi Centers for Disease Control (CDC) between January 2012 and December 2016 were included in this study. The inclusion criteria were as follow: (1) HIV positive, (2) aged at least 15 years, (3) had a CD4^+^ T-cell count during diagnosis (it was defined as the first CD4^+^ T-cell count detection within 3 months after diagnosis), (4) were ART-naïve when the first CD4^+^ T-cell count was detected. This study excluded HIV-1 infected patients who had no record of CD4^+^ T-cell count, had the first CD4^+^ T-cell count detection longer than 3 months after diagnosis, and were on ART before the CD4^+^ T-cell count was detected.

### Study design

A cross-sectional study was conducted to investigate the prevalence of late presentation and advanced HIV disease among patients with newly diagnosed HIV/AIDS and the influencing factors associated with late presentation and advanced HIV disease. Demographic or socioeconomic data, including gender, age, region, marital status, occupation, ethnic, educational attainment, and HIV transmission route, as well as clinical data, such as CD4^+^ T-cell count at diagnosis, year of HIV diagnosis, and reason for HIV testing, were collected from the records of HIV surveillance system and used for subsequent analyses.

### Definitions

CD4^+^ T-cell counts were determined by flow cytometry. According to a consensus definition as presented by the European Late Presenter Consensus working group [23], late presentation was defined as, a patient diagnosed with the first CD4^+^ T-cell count < 350/mm^3^, or a patient with a AIDS-defining illness regardless of CD4^+^ T-cell count during diagnosis. Besides, advanced HIV disease was defined as a patient with a CD4^+^ T-cell count < 200/mm^3^, or a patient with an AIDS-defining illness regardless of CD4^+^ T-cell count during diagnosis.

### Statistical analysis

The trends of late presentation and advanced HIV disease were analyzed using the Chi square test. The risk factors associated with late presentation and advanced HIV disease were analyzed by univariate and multivariate logistic regression analyses. The univariate analysis explored variables (attributes) one by one. Variables could be either categorical or numerical. The multivariate analysis was based on the statistical principle of multivariate statistics, which involved observation and analysis of more than one statistical outcome variable at a time. A P-value less than 0.05 was considered statistically significant. Statistical analyses were performed using the SPSS16.0 software (IBM, NY, USA).

## Results

### Characteristics of late presentation and advanced HIV disease

**Fig. 1:**
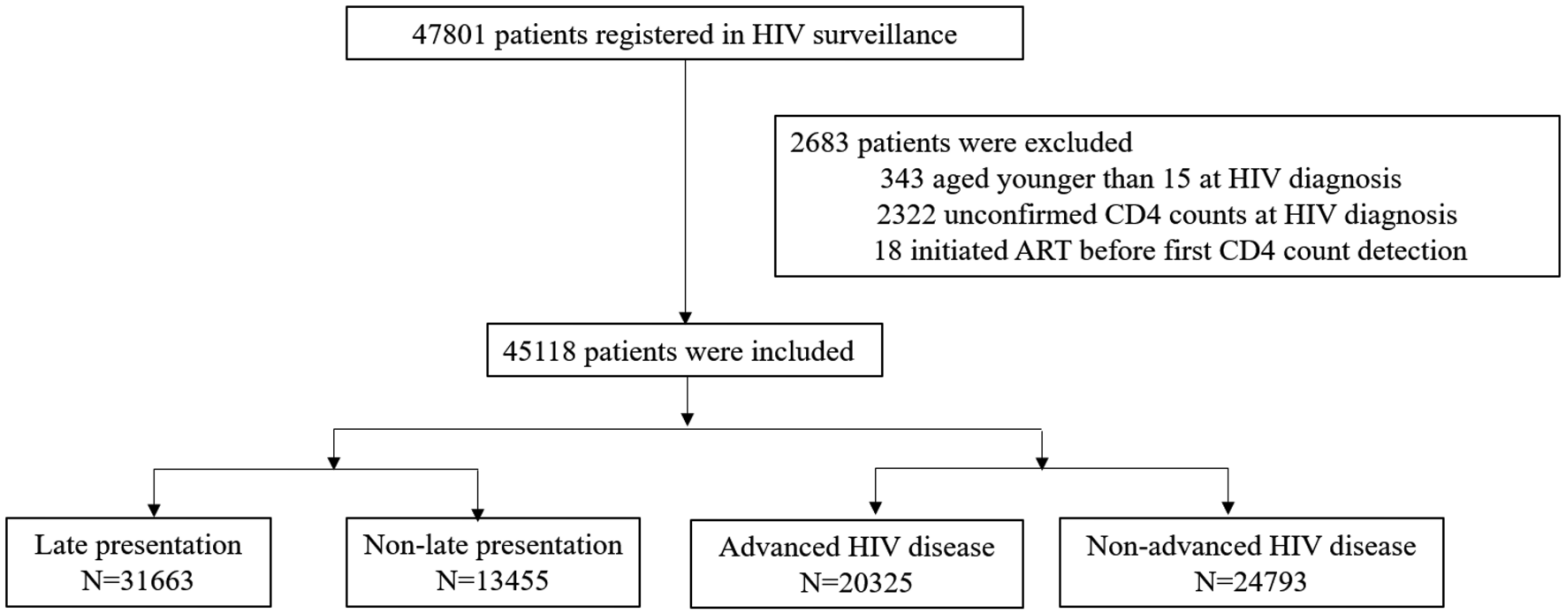
Chart of the inclusion and exclusion criteria in this study

A total of 47,801 patients with newly diagnosed HIV registered in the HIV surveillance system of Guangxi CDC between 2012 and 2016. Among them, 45,118 patients met the inclusion criteria with no missing data regarding the CD4^+^ T-cell count in 3 months after diagnosis and those aged 15 years or older during diagnosis (Fig. 1). Their characteristics are summarized in Table 1. The overall median CD4^+^ T-cell count was 228/mm^3^ [interquartile range (IQR): 78–383]. In the study population, 70.2% (31,663/45,118) were diagnosed with late presentation with a mean age of 49.8 years, and 45.0% (20,325/45,118) were diagnosed with advanced HIV disease with a mean age of 50.3 years (Table 1). Higher percentages of late presentation and advanced HIV disease were found in men (late presentation/advanced HIV disease: 73.6%/77.6%), those aged more than 50 years (late presentation/advanced HIV disease: 48.9%/49.3%), migrant workers (late presentation/advanced HIV disease: 77.5%/79.5%), married (late presentation/advanced HIV disease: 62.2%/61.0%), Han ethnicity (late presentation/advanced HIV disease: 62.6%/63.5%), primary/no education (late presentation/advanced HIV disease: 88.7%/90.0%), transmission by heterosexual sex (late presentation/advanced HIV disease: 94.9%/96.1%), and those diagnosed in hospital (late presentation/advanced HIV disease: 45.1%/52.3%) (Table 1).

**Table 1:** The demographic and sociological features of the patients

Characteristics at HIV diagnosis	Late presentation N (%)	Non-late presentation N (%)	Advanced HIV disease N (%)	Non-advanced HIV disease N (%)
Year of diagnosis				
2012	6873 (21.7)	2454 (18.2)	4535 (22.3)	4792 (19.4)
2013	6833 (21.6)	3140 (23.4)	4239 (20.9)	5734 (23.1)
2014	5976 (18.9)	2851 (21.2)	3848 (18.9)	4979 (20.1)
2015	5864 (18.5)	2681 (19.9)	3677 (18.1)	4868 (19.6)
2016	6117 (19.3)	2329 (17.3)	4026 (19.8)	4420 (17.8)
Gender				
Male	23,307 (73.6)	8721 (64.8)	15,766 (77.6)	16,262 (65.6)
Female	8356 (26.4)	4734 (35.2)	4559 (22.4)	8531 (34.4)
Age (years)				
15–30	3533 (11.2)	3216 (23.9)	1812 (8.9)	4937 (19.9)
31–50	12,640 (39.9)	5360 (39.8)	8488 (41.8)	9512 (38.4)
> 50	15,490 (48.9)	4879 (36.3)	10,025 (49.3)	10,344 (41.7)
Occupation				
Migrant workers	24,546 (77.5)	9281 (69.0)	16,154 (79.5)	17,673 (71.3)
Other	7117 (22.5)	4174 (31.0)	4171 (20.5)	7120 (28.7)
Marital status				
Married	19,686 (62.2)	8184 (60.8)	12,402 (61.0)	15,468 (62.4)
Divorced or widowed	6329 (20.0)	1965 (14.6)	4281 (21.1)	4013 (16.2)
Single	5648 (17.8)	3306 (24.6)	3642 (17.9)	5312 (21.4)
Ethnic				
Han	19,817 (62.6)	8215 (61.1)	12,906 (63.5)	15,126 (61.0)
Zhuang	10,565 (33.4)	4619 (34.3)	6627 (32.6)	8557 (34.5)
Others	1281 (4.0)	621 (4.6)	792 (3.9)	1110 (4.5)
Level of education				
Primary/no education	28,094 (88.7)	11,212 (83.3)	18,295 (90.0)	21,011 (84.7)
Secondary/university	3569 (11.3)	2243 (16. 7)	2030 (10.0)	3782 (15.3)
HIV transmission group				
Heterosexual sex	30,042 (94.9)	11,952 (88.8)	19,526 (96.1)	22,468 (90.6)
PWID	597 (1.9)	480 (3.6)	347 (1.7)	730 (3.0)
MSM	836 (2.6)	935 (7.0)	332 (1.6)	1439 (5.8)
Other	188 (0.6)	88 (0.6)	120 (0.6)	156 (0.6)
Reason for HIV testing				
STI clinics	1001 (3.2)	404 (3.0)	618 (3.0)	787 (3.2)
Premarital/pregnancy screening	1283 (4.1)	1308 (9.7)	460 (2.3)	2131 (8.6)
Medical examination	83 (0.3)	100 (0.7)	35 (0.2)	148 (0.6)
Penitentiary	585 (1.8)	600 (4.5)	264 (1.3)	921 (3.7)
HIV + couple or partner	1659 (5.2)	1003 (7.5)	773 (3.8)	1889 (7.6)
Pre-surgery	2409 (7.6)	1128 (8.4)	1393 (6.8)	2144 (8.6)
VCT	8556 (27.0)	4311 (32.0)	5204 (25.6)	7663 (30.9)
Hospital	14,279 (45.1)	3532 (26.3)	10,620 (52.3)	7191 (29)
Blood testing of the blood receivers^a^	320 (1.0)	76 (0.6)	225 (1.1)	171 (0.7)
Other	1488 (4.7)	993 (7.4)	733 (3.6)	1748 (7.1)

*MSM* men who have sex with men, *PWID* people who use drugs, *STI* sexually transmitted infection, *VCT* voluntary counseling and testing

^a^Blood testing of the blood receivers: HIV testing among patients who were going to receive blood transfusion

### Percentages of late presentation and advanced HIV disease by the transmission group and gender

**Fig. 2:**
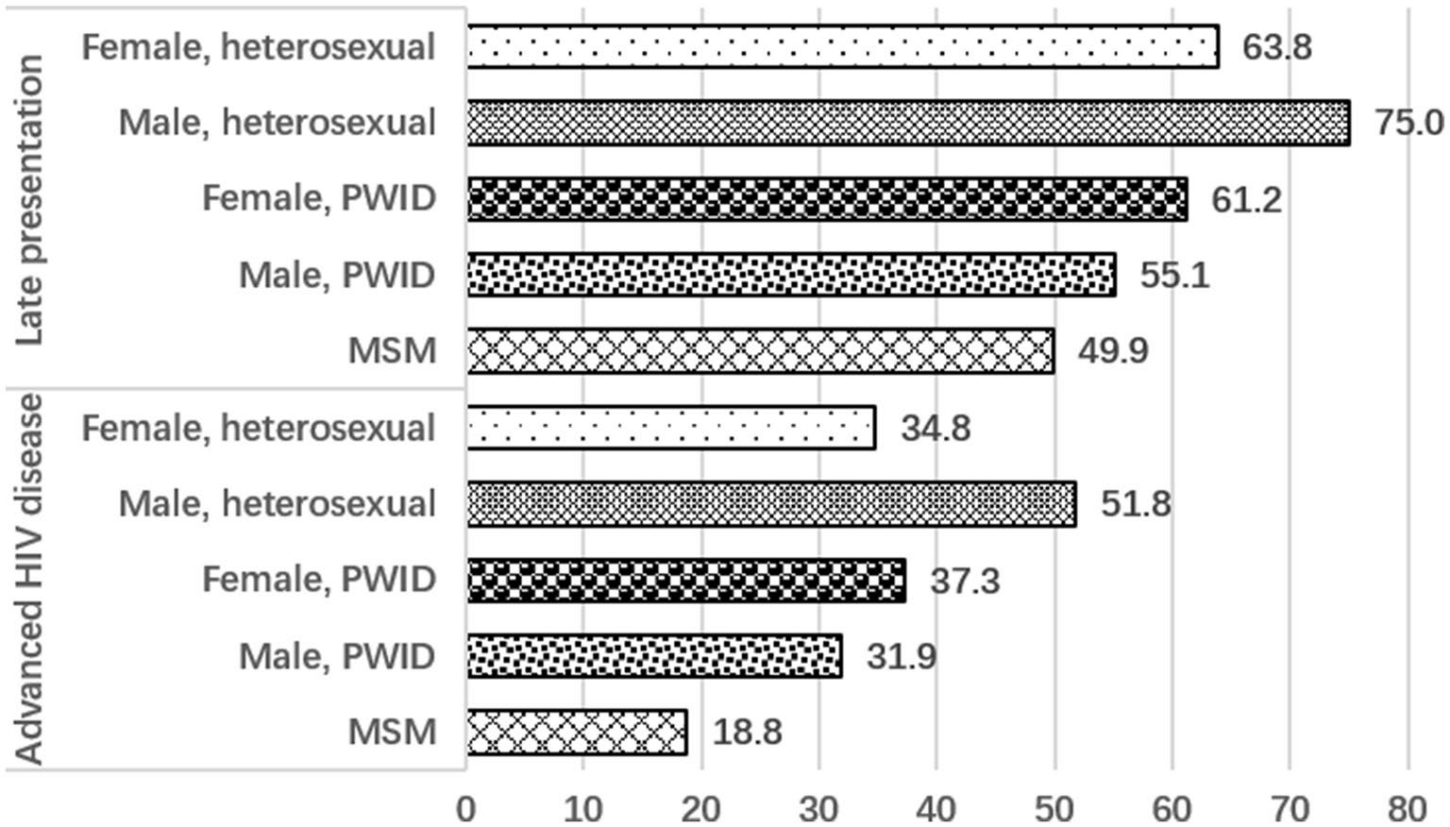
Percentages of HIV late presentation and advanced HIV disease for 2012–2016, by transmission groups (*MSM* men who have sex with men, *PWID* people who use drugs) and gender

Percentages of late presentation and advanced HIV disease by the main transmission group and gender are shown in Fig. 2. Overall, compared with the groups of heterosexuals and people who use drugs (PWID), the men who have sex with men (MSM) group showed the lowest percentages of late presentation and advanced HIV disease (late presentation: 49.9%; advanced HIV disease: 18.8%). The highest percentages of late presentation and advanced HIV disease were found in male heterosexuals (late presentation: 75.0%; advanced HIV disease: 51.7%). Among PWID, women had higher percentages of late presentation and advanced HIV disease (late presentation: 61.2%; advanced HIV disease: 37.3%) compared with men (late presentation: 55.1%; advanced HIV disease: 31.9%) (Fig. 2).

### Trends of late presentation and advanced HIV disease

**Fig. 3:**
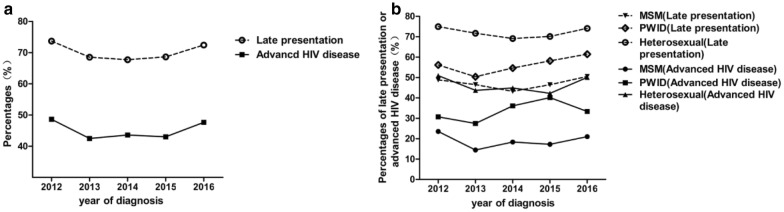
Changes of late presentation and advanced HIV disease by year of diagnosis (**a**) and transmission groups (*MSM* men who have sex with men, *PWID* people who use drugs) (**b**)

The trends of late presentation and advanced HIV disease during 2012–2016 by transmission groups (MSM, heterosexual, and PWID) are presented in Fig. 3. Overall, the percentage of patients with late presentation or advanced HIV disease was relatively stable over time. No significant difference was found in the trend test (late presentation: χ^2^ = 3.14, P = 0.07; advanced HIV disease: χ^2^ = 0.92, P = 0.34) (Fig. 3a). The trend analysis of late presentation or advanced HIV disease by main transmission groups (MSM, heterosexual and PWID) yielded similar results (no significant difference, P > 0.05) (Fig. 3b).

### Factors associated with late presentation

As shown in Table 2, in a univariate analysis, patients with late presentation were significantly associated with male sex, older age, migrant workers, divorced or widowed, primary/no education,  blood testing of blood receivers and diagnosed with HIV in hospital and STI clinics. These variables were further included in the multivariate analysis (Table 2), Which showed that late presentation significantly increased with age during diagnosis [adjusted odds ratio (aOR; 95% confidence interval (CI) for 30–50 years old vs. 15–30 years old: 1.68 (1.58–1.79); > 50 years old vs. < 15–30 years old: 1.97 (1.85–2.11)]. Men had a stronger association with the probability of late presentation [aOR: 1.43; 95% CI (1.36–1.50)]. Patients transmitted by heterosexual sex [aOR: 2.11; 95% CI (1.90–2.34)], PWID [aOR: 1.55; 95% CI (1.30–1.84)], or other transmissions including contaminated blood transfusion [aOR: 2.22; 95% CI (1.67–2.95)], had a higher risk of late presentation compared with MSM. Hospital and blood testing of the blood receivers had an increased risk of late presentation compared with voluntary counseling and testing (VCT), with an aOR of 1.74 (95% CI 1.65–1.84) and 1.75 (95% CI 1.36–2.26) respectively. Patients who were diagnosed HIV for medical examination [aOR: 0.47; 95% CI (0.35–0.63)], penitentiary [aOR: 0.52; 95% CI (0.45–0.59)], or premarital/pregnancy screening [aOR: 0.63; 95% CI (0.58–0.69)], had a significantly greater probability for early diagnosis (Table 2).

**Table 2:** Risk factors associated with late presentation (logistic regression model), Guangxi, 2012–2016

Characteristics at HIV diagnosis	cOR (95% CI)	P	aOR (95% CI)	P
Gender				
Male	1.51 (1.45–1.58)	< 0.001	1.43 (1.36–1.50)	< 0.001
Female	Reference		Reference	
Age (years)				
15–30	Reference		Reference	
31–50	2.15 (2.03–2.27)	< 0.001	1.68 (1.58–1.79)	< 0.001
> 50	2.89 (2.73–3.06)	< 0.001	1.97 (1.85–2.11)	< 0.001
Occupation				
Migrant workers	1.55 (1.48–1.62)	< 0.001		
Other	Reference			
Marital status				
Married	1.41 (1.34–1.48)	< 0.001		
Divorced or widowed	1.89 (1.76–2.02)	< 0.001		
Single	Reference			
Ethnic				
Han	1.17 (1.06–1.29)	0.002		
Zhuang	1.11 (1.00–1.23)	0.047		
Others	Reference			
Level of education				
Primary/no education	1.58 (1.49–1.67)	< 0.001		
Secondary/university	Reference			
HIV transmission group				
Heterosexual sex	2.81 (2.56–3.10)	< 0.001	2.11 (1.90–2.34)	< 0.001
PWID	1.39 (1.20–1.62)	< 0.001	1.55 (1.30–1.84)	< 0.001
Other	2.40 (1.82–3.13)	0.004	2.22 (1.67–2.95)	< 0.001
MSM	Reference		Reference	
Reason for HIV testing				
STI clinics	1.25 (1.11–1.41)	< 0.001	1.21 (1.07–1.37)	0.002
Premarital/pregnancy screening	0.49 (0.45–0.54)	< 0.001	0.63 (0.58–0.69)	< 0.001
Medical examination	0.42 (0.31–0.56)	< 0.001	0.47 (0.35–0.63)	< 0.001
Penitentiary	0.49 (0.44–0.55)	< 0.001	0.52 (0.45–0.59)	< 0.001
HIV + couple or partner	0.83 (0.76–0.91)	< 0.001	0.83 (0.76–0.91)	<0.001
Pre-surgery	1.08 (0.99–1.17)	0.071	0.94 (0.87–1.02)	0.152
Hospital	2.04 (1.93–2.15)	< 0.001	1.74 (1.65–1.84)	< 0.001
Blood testing of the blood receivers^a^	2.12 (1.65–2.73)	< 0.001	1.75 (1.36–2.26)	< 0.001
Other	0.85 (0.78–0.93)	< 0.001	0.70 (0.64–0.76)	< 0.001
VCT	Reference		Reference	

*cOR* crude odds ratio, *aOR* adjusted odds ratio, *MSM* men who have sex with men, *PWID* people who use drugs, *STI* sexually transmitted infection, *VCT* voluntary counseling and testing

^a^Blood testing of the blood receivers: HIV testing among patients who were going to receive blood transfusion

### Factors associated with advanced HIV disease

As shown in Table 3, in a univariate analysis, patients with advanced HIV disease were significantly associated with male sex, older age, migrant workers, divorced or widowed, primary/no education, blood testing of blood receivers and diagnosed HIV in hospital and STI clinics. These variables were further included in the multivariate analysis (Table 3). The results showed that the patients aged 31 to 50 years [aOR: 1.68; 95% CI (1.56–1.80)], and those aged older than 50 years [aOR: 1.51; 95% CI (1.40–1.62)], had a higher occurrence of advanced HIV disease compared with those aged 15 to 30 years. Compared with female patients, male patients had a higher aOR of advanced HIV disease [aOR: 1.66; 95% CI (1.58–1.74)]. Compared with MSM, all other HIV transmission groups were more likely to be associated with advanced HIV disease, especially in PWID [(aOR 2.23; 95% CI (1.83–2.71)] and in heterosexuals [(aOR 2.86; 95% CI (2.51–3.27)]. Tested HIV in hospital [aOR: 1.85; 95% CI (1.76–1.94)] was found to be an independent relative factor for advanced HIV disease among the factors for HIV testing. Advanced HIV disease was also more common in blood testing of the blood receivers (aOR 1.58, CI 1.29 to 1.94). In addition, diagnosed HIV for premarital/pregnancy screening [aOR: 0.36; 95% CI (0.33–0.41)], medical examination [aOR: 0.39; 95% CI (0.27–0.56)], penitentiary [aOR: 0.37; 95% CI (0.32–0.44)], HIV-positive couple or partner [aOR: 0.65; 95% CI (0.59–0.72)], and pre-surgery [aOR: 0.83; 95% CI (0.77–0.90)] were protective factors for the occurrence of advanced HIV disease.

**Table 3:** Risk factors associated with advanced HIV disease (logistic regression model), Guangxi, 2012–2016

Characteristics at HIV diagnosis	cOR (95% CI)	P	aOR (95% CI)	P
Gender				
Male	1.81 (1.74–1.90)	< 0.001	1.66 (1.58–1. 74)	< 0.001
Female	Reference		Reference	
Age (years)				
15–30	Reference		Reference	
31–50	2.43 (2.29–2.59)	< 0.001	1.68 (1.56–1.80)	< 0.001
> 50	2.64 (2.49–2.81)	< 0.001	1.51 (1.40–1.62)	< 0.001
Occupation				
Migrant worker	1.56 (1.49–1.63)	< 0.001	1.17 (1.11–1.23)	< 0.001
Other	Reference		Reference	
Marital status				
Married	1.17 (1.11–1.23)	< 0.001	0.89 (0.83–0.94)	< 0.001
Divorced or widowed	1.556 (1.465–1.653)	< 0.001	1.08 (1.00–1.16)	0.04
Single	Reference		Reference	
Ethnic				
Han	1.20 (1.09–1.31)	< 0.001		
Zhuang	1.09 (0.99–1.20)	0.10		
Others	Reference			
Level of education				
Primary/no education	1.62 (1.53–1.72)	< 0.001	1.22 (1.14–1.30)	< 0.001
Secondary/university	Reference		Reference	
HIV transmission group				
Heterosexual	3.77 (3.34–4.25)	< 0.001	2.86 (2.51–3.27)	< 0.001
PWID	2.06 (1.73–2.45)	< 0.001	2.23 (1.83–2.71)	< 0.001
Other	3.33 (2.56–4.35)	< 0.001	2.94 (2.21–3.91)	< 0.001
MSM	Reference		Reference	
Reason for HIV testing				
STI clinics	1.16 (1.04–1.29)	0.01	1.08 (0.96–1.21)	0.19
Premarital/pregnancy screening	0.32 (0.29–0.35)	< 0.001	0.36 (0.33–0.41)	< 0.001
Medical examination	0.35 (0.24–0.50)	< 0.001	0.39 (0.27–0.56)	< 0.001
Penitentiary	0.42 (0.37–0.49)	< 0.001	0.37 (0.32–0.44)	< 0.001
HIV + couple or partner	0.60 (0.55–0.66)	< 0.001	0.65 (0.59–0.72)	< 0.001
Pre-surgery	0.96 (0.89–1.03)	0.25	0.83 (0.77–0.90)	< 0.001
Hospital	2.18 (2.08–2.28)	< 0.001	1.85 (1.76–1.94)	< 0.001
Blood testing of the blood receivers^a^	1.948 (1.54–2.37)	< 0.001	1.58 (1.29–1.94)	< 0.001
Other	0.74 (0.68–0.80)	< 0.001	0.54 (0.49–0.60)	< 0.001
VCT	Reference		Reference	

*cOR* crude odds ratio, *aOR* adjusted odds ratio, *MSM* men who have sex with men, *PWID* people who use drugs, *STI* sexually transmitted infection, *VCT* voluntary counseling and testing

^a^Blood testing of the blood receivers: HIV testing among patients who were going to receive blood transfusion

## Discussion

This novel study investigated the prevalence of late presentation and advanced HIV disease in Southwestern China. One strength of this study was its large sample size. Further, the completeness of the data was high, because more than 94% of the newly diagnosed patients were included in this study, and also the important information, including CD4 T-cell counts during diagnosis or the first entry into care and AIDS-defining illnesses, was available.

The study showed that the local prevalence of late presentation reached up to ~ 70% in recent years, indicating that late diagnosis is a serious problem in Guangxi, China The prevalence of late presentation in Guangxi not only is higher than those in other countries [24, 25], but also higher than those in other regions in China, including Zhejiang province [26] and Guangzhou City [27]. Besides, the prevalence of advanced HIV disease in Guangxi is higher compared with national level in China (42.1% vs. 35.5%) [10]. Late presentation is associated with an increased rate of AIDS/deaths, particularly in the first year after HIV diagnosis [23]. Therefore, a high prevalence of late presentation and advanced HIV disease may be an important factor for the high mortality among patients with HIV/AIDS in Guangxi. Reducing late HIV diagnosis and advanced HIV disease is considered a public health priority and continues to be a great challenge in most countries [28, 29]. To achieve this goal, the US CDC and the US Preventive Services Task Force newly recommended one-time HIV testing for persons aged 15–65 years as part of routine health care [30, 31].

This study revealed that men were associated with late presentation, which was consistent with the results of previous studies. A meta-analysis which including 32 studies, revealed that the pooled aOR of men with advanced HIV disease and late presentation compared with women was 1.73 and 1.38, respectively [32]. One possible reason was that HIV-infected women generally experienced a slower disease development compared with men, which was corroborated by the fact that women tended to have higher CD4^+^ lymphocyte counts compare with men with similar infection time [33]. Nevertheless, two other studies from Belgium and North-East Scotland [34, 35] suggested that women were more likely diagnosed late. This discrepancy might be contributed to the fear of stigma and discrimination, being a significant issue particularly among women in certain areas in the world.

Older age was also found to be a factor associated with late presentation in this study, which was similar to the results of other previous studies [36, 37]. This might be due to several reasons. The HIV symptoms in older people were misjudged as other illnesses for being older [38, 39], or the elderly were hard to be a target of HIV prevention efforts [40]. Psychological factors, such as depression, associated with delayed diagnosis and late testing [41], are more common in older adults, which may also hinder access to health care [42]. The study indicated that older age was also associated with advanced HIV disease, which was different from the findings of some similar studies [43–45].

In this study, PWID and heterosexuals were more likely to be associated with late presentation and advanced HIV disease compared with MSM. The reason, possibly, was that the access or barriers of HIV testing for different risk populations might be different. Most of PWID were reluctance to access the health care system, while they were asymptomatic [46, 47]. However, MSMs were more likely to have an HIV testing due to various encouragement strategies, such as opt-out HIV testing (tests are routinely offered to all patients) at STI clinics and the encouragement of high-risk MSMs for HIV testing every 6 months [48]. For heterosexuals, effective HIV testing strategies are lacking. Hence, a more forward-looking proposal of HIV testing in different health care settings is urgently needed to reduce the high rates of late diagnosis.

Diagnosed HIV in hospital and those that had HIV testing before accepting blood transfusion were two relatively strong factors associated with late presentation and advanced HIV disease, suggesting that patients didn’t visit a doctor until the clinical symptoms appeared and treatment was initiated at a later disease stage. The other possible explanations could be that the clinical manifestations lack specificity, contributing to the missed diagnosis of HIV infection by health care professionals. Health care professionals should play an important role in recommending HIV testing in the presence of AIDS defining diseases as well as for the specific HIV indicator conditions [49]. On the contrary, the higher CD4 lymphocyte count at presentation was found among patients with an HIV-positive partner or those who were diagnosed by premarital/pregnancy screening, medical examination, penitentiary, pre-surgery and VCT, indicating that routine HIV testing is an effective measure to reduce late diagnosis. A recent report has shown that the lack of routine HIV testing is a general health challenge, associated with a poor medical level in rural areas [50]. Hence, efforts should be made to detect patients with HIV/AIDS promptly.

This study had several limitations. First, participants of this study were from one province of China, thus leading to a selection bias. Nevertheless, the large sample size and multicenter study in essence (the subjects came from all counties and cities across Guangxi province) could partially reduce the bias. Second, the details of the category of AIDS defining illness were unavailable. Third, variables used in this study were somewhat limited because this study was actually a retrospective cross-sectional investigation, and some influencing factors might have been missed. Further study focusing on the association of knowledge, attitude, and practice of patients toward HIV/AIDS and the clinical symptoms of patients with late diagnosis, should be conducted to better identify the influencing factors and control late diagnosis.

## Conclusions

In conclusion, late diagnosis was quite common among patients with newly diagnosed HIV/AIDS in Guangxi, China, during 2012–2016, which is a challenge for the prevention and control of HIV/AIDS. Given the fact that late diagnosis in Guangxi has not shown a decreasing trend in recent years, targeted programs should be urgently designed to reduce HIV late diagnosis.

## Abbreviations

UNAIDS: the Joint United Nations Programme on HIV/AIDS
ART: antiretroviral therapy
HIV: human immunodeficiency virus
AIDS: acquired immune deficiency syndrome
MSM: men who have sex with men
PWID: people who use drug
CDC: Centers for Disease Control
VCT: voluntary counseling and testing
IQR: interquartile range
aOR: adjusted odds ratio
STI: sexually transmitted infection
